# Downregulation of miR-194-5p induces paclitaxel resistance in ovarian cancer cells by altering MDM2 expression

**DOI:** 10.18632/oncotarget.26586

**Published:** 2019-01-18

**Authors:** Koji Nakamura, Kenjiro Sawada, Mayuko Miyamoto, Yasuto Kinose, Akihiko Yoshimura, Kyoso Ishida, Masaki Kobayashi, Aasa Shimizu, Erika Nakatsuka, Kae Hashimoto, Seiji Mabuchi, Tadashi Kimura

**Affiliations:** ^1^ Department of Obstetrics and Gynecology, Osaka University Graduate School of Medicine, Suita, Osaka, 5650871, Japan; ^2^ Department of Molecular Oncology, H. Lee Moffitt Cancer Center & Research Institute, Tampa, FL, 33612, USA; ^3^ Penn Ovarian Cancer Research Center, Perelman School of Medicine, University of Pennsylvania Perelman School of Medicine, Biomedical Research Building II/III, Philadelphia, PA, 19104, USA

**Keywords:** microRNA, miR-194-5p, paclitaxel resistance, MDM2, ovarian cancer

## Abstract

Paclitaxel is a first-line drug for treating epithelial ovarian cancer (EOC). However, prognosis for patients with advanced stage cancer remains poor due to primary or acquired drug resistance. Therefore, overcoming chemoresistance is one of the greatest challenges in treating EOC. In this study, we identified microRNAs (miRNA) that regulate paclitaxel resistance and tested their potential utility as therapeutic targets. Paclitaxel-resistant cell lines were established using two EOC cell lines: SKVO3ip1 and HeyA8. miRNA PCR arrays showed that miR-194-5p was downregulated in paclitaxel-resistant cells. Forced expression of miR-194-5p resensitized resistant cells to paclitaxel. Conversely, miR-194-5p inhibition induced paclitaxel resistance in parental cells. *In silico* analysis and luciferase reporter assay revealed that *MDM2* is a direct target of miR-194-5p. *MDM2* was upregulated in paclitaxel resistant cells compared with parental cells. *MDM2* inhibition also resensitized resistant cells to paclitaxel and forced MDM2 induced paclitaxel resistance in parental cells. miR-194-5p induced p21 upregulation and G1 phase arrest in resistant cells by downregulating *MDM2*. Furthermore, a public database showed that high *MDM2* expression was associated with a shorter progression-free survival in EOC patients treated with paclitaxel. Collectively, our results show that restoring miR-194-5p expression resensitizes EOCs to paclitaxel, and this may be exploited as a therapeutic option.

## INTRODUCTION

Ovarian cancer is the most lethal gynecological malignancy and the fifth leading cause of cancer-related death in women in developed countries [1]. The majority of ovarian cancers are epithelial ovarian cancer (EOC) and the standard therapy for advanced EOC involves both cytoreductive surgery and intensive chemotherapy, with combination paclitaxel and platinum as the standard adjuvant chemotherapy regimen [2]. However, despite these multimodal treatments, patients eventually face recurrence and the 5-year survival rate remains, at most, 30% and has not improved during the past 20 years [1]. The major cause of recurrence is acquired resistance to chemotherapeutic agents. Therefore, it is urgent to elucidate the underlying mechanisms that lead to chemoresistance to improve the efficacy of therapeutic strategies.

The combination of taxane and platinum has remained the first-line chemotherapy for ovarian cancer treatment for the past 20 years [2, 3]. Paclitaxel is the most widely used taxane and is one of key drugs for treating EOC. Thus, paclitaxel resistance determines the fate of patients with EOC. Over the past decade, considerable progress has been made toward understanding the mechanisms that underlie paclitaxel resistance; however, the molecular mechanisms underlying this resistance are still not completely elucidated. microRNAs (miRNAs) are endogenous non-coding RNAs approximately 22-nucleotides in length that play important roles in a wide range of physiological and pathophysiological processes by regulating gene expression by binding to the 3′-untranslated region (UTR) of target genes. More than 50% of miRNA target genes are located in cancer-associated genomic regions or in fragile sites, suggesting that miRNAs are deeply involved in cancer pathogenesis [4]. Emerging evidence shows that chemoresistance is also affected by dysregulation of miRNAs in a variety of cancer types [5]. While several miRNAs have been reported to be associated with paclitaxel resistance, our understanding of the role of miRNAs in this process still remains limited.

Herein, we identified miRNAs that display altered expression patterns after the acquisition of paclitaxel resistance in EOC cells. We found that miR-194-5p is one of the downregulated miRNAs in paclitaxel-resistant cells and restoring miR-194-5p significantly attenuated paclitaxel resistance. Further, we identified *MDM2* as a direct target gene of miR-194-5p and revealed the role of MDM2 and miR-194-5p by cell cycle analyses.

## RESULTS

### Establishment of paclitaxel-resistant ovarian cancer cell lines

**Figure 1:**
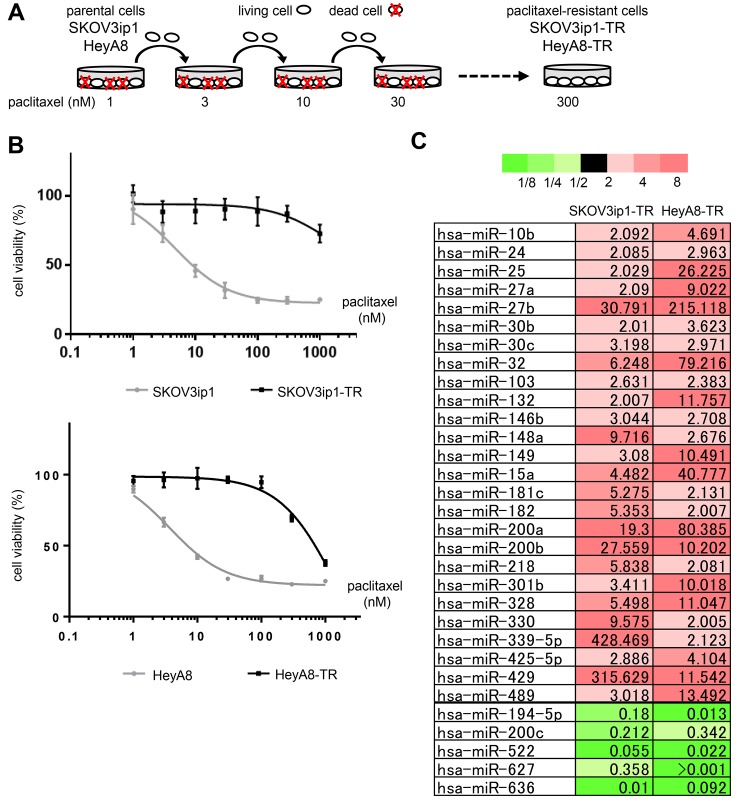
miR-194-5p is downregulated in paclitaxel-resistant ovarian cancer cell lines (**A**) Scheme for the establishment of two paclitaxel-resistant sublines, SKOV3ip1-TR and HeyA8-TR, derived from SKOV3ip1 and HeyA8, respectively. These cells were exposed to stepwise increases of paclitaxel until a concentration of 300 nmol/L. (**B**) *In vitro* survival assay of ovarian cancer cell lines upon paclitaxel treatment. Growth inhibitory effects of paclitaxel treatment were determined using an MTS assay. Experiments were performed in triplicate. Data are represented as mean ± SE and are obtained from three independent experiments. (**C**) miRNA microarray. List of miRNAs that exhibited increased (> 2-fold, red columns) or decreased (< 0.5-fold, green columns) expression in both paclitaxel-resistant sublines compared with their corresponding parental cell lines.

First, we established paclitaxel-resistant ovarian cancer cell lines from two different high-grade serous ovarian cancer cell lines, SKOV3ip1 and HeyA8, by repeated exposure to stepwise increases of paclitaxel (Figure 1A). The two established sublines, named SKOV3ip1-TR and HeyA8-TR, respectively, showed higher IC_50_ values for paclitaxel (SKOV3ip1-TR: > 1000 nM, HeyA8-TR: 647 nM) than their parental cells (SKOV3ip1: 4.76 nM, HeyA8: 3.74 nM) (Figure 1B).

### miR-194-5p is downregulated in paclitaxel-resistant ovarian cancer cell lines

To analyze the involvement of miRNAs during the acquisition of paclitaxel resistance, Taqman miRNA arrays were performed using SKOV3ip1, HeyA8, and their respective paclitaxel-resistant sublines. In both paclitaxel-resistant cell lines, miR-194-5p, miR-200c, miR-522, miR-627, and miR-633 were downregulated (i.e., the level of expression in paclitaxel-resistant cell lines was < 0.5-fold of that in the parental cell lines), and 26 miRNAs were upregulated (i.e., the level of expression in paclitaxel-resistant cell lines was > 2.0-fold of that in the parental cell lines) (Figure 1C). Among these downregulated miRNAs, recent studies have suggested that miR-194-5p has a potential role as a tumor suppressor in several types of cancer [6, 7] and that it is downregulated in paclitaxel-resistant ovarian cancer tissue [8]. Thus, we focused on the mechanism underlying miR-194-5p action during the acquisition of paclitaxel resistance.

### miR-194-5p modulates sensitivity to paclitaxel

**Figure 2:**
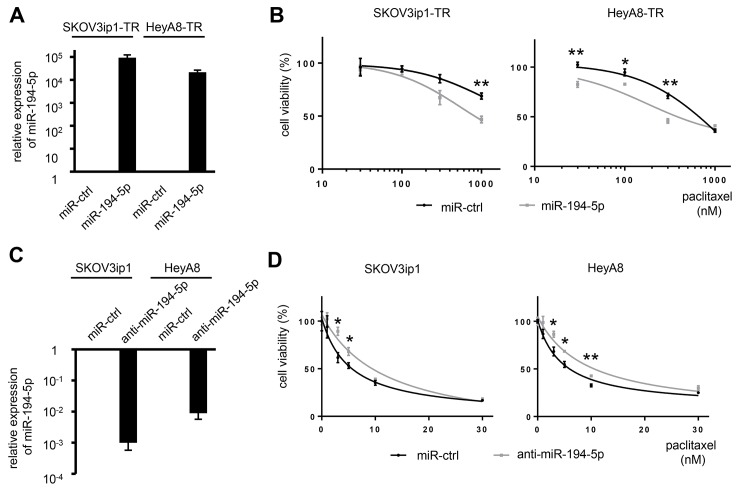
miR-194-5p modulates sensitivity to paclitaxel in ovarian cancer cell lines (**A**) miRNA qRT-PCR. Cells were transfected with pre-miR-194-5p (miR-194-5p) or control miR (miR-ctrl). Twenty-four hours later, expression of miR-194-5p relative to RNU6B expression was calculated using the 2^-ΔΔCT^ method. Relative fold differences are presented. (**B**) MTS assay. Twenty-four hours after transfection with miR-194-5p or control miR-ctrl, cells were treated with paclitaxel for 72 hours and cell viability was assessed. Cell viability is shown relative to that in paclitaxel-free conditions. (**C**) miRNA qRT-PCR. Cells were transfected with anti-miR-194-5p or miR-ctrl for 24 hours. (**D**) MTS assay. As described in B, cells were transfected with anti-miR-194 or miR-ctrl and cell viability was assessed. Experiments were performed in triplicate. Data are represented as mean ± SE and are obtained from three independent experiments. ^*^P < 0.05; ^**^P < 0.01.

To determine whether miR-194-5p is associated with paclitaxel resistance, cell viability assays were performed by either restoring or silencing miR-194 alongside paclitaxel treatment. SKOV3ip1-TR and HeyA8-TR cells were transfected with miR-194-5p precursor or control miRNA. SKOV3ip1 and HeyA8 cells were transfected with a miR-194-5p antagonist or control miRNA. The overexpression or inhibition of miR-194-5p in these cell lines was confirmed by miRNA quantitative RT-PCR (Figure 2A, 2C). miR-194-5p-transfected paclitaxel-resistant cells were more sensitive to paclitaxel than their corresponding controls. IC_50_ values for paclitaxel in SKOV3ip1-TR cells treated with miR-ctrl and miR-194-5p were > 1000 nM and 816 nM, respectively. The IC_50_ for paclitaxel in HeyA8-TR cells treated with miR-ctrl and miR-194-5p were 634 nM and 440 nM, respectively (Figure 2B). Conversely, anti-miR-194-5p-transfected parental cells showed more resistance to paclitaxel than the corresponding controls. IC_50_ values for paclitaxel in SKOV3ip1 cells were 5.22 nM (miR-ctrl) and 11.6 nM (anti-miR-194-5p), respectively, and they were 4.67 nM (miR-ctrl) and 9.06 nM (anti-miR-194-5p), respectively in HeyA8 cells (Figure 2D). These results indicated that miR-194-5p modulates paclitaxel sensitivity.

### MDM2 is a direct target of miR-194-5p

**Figure 3:**
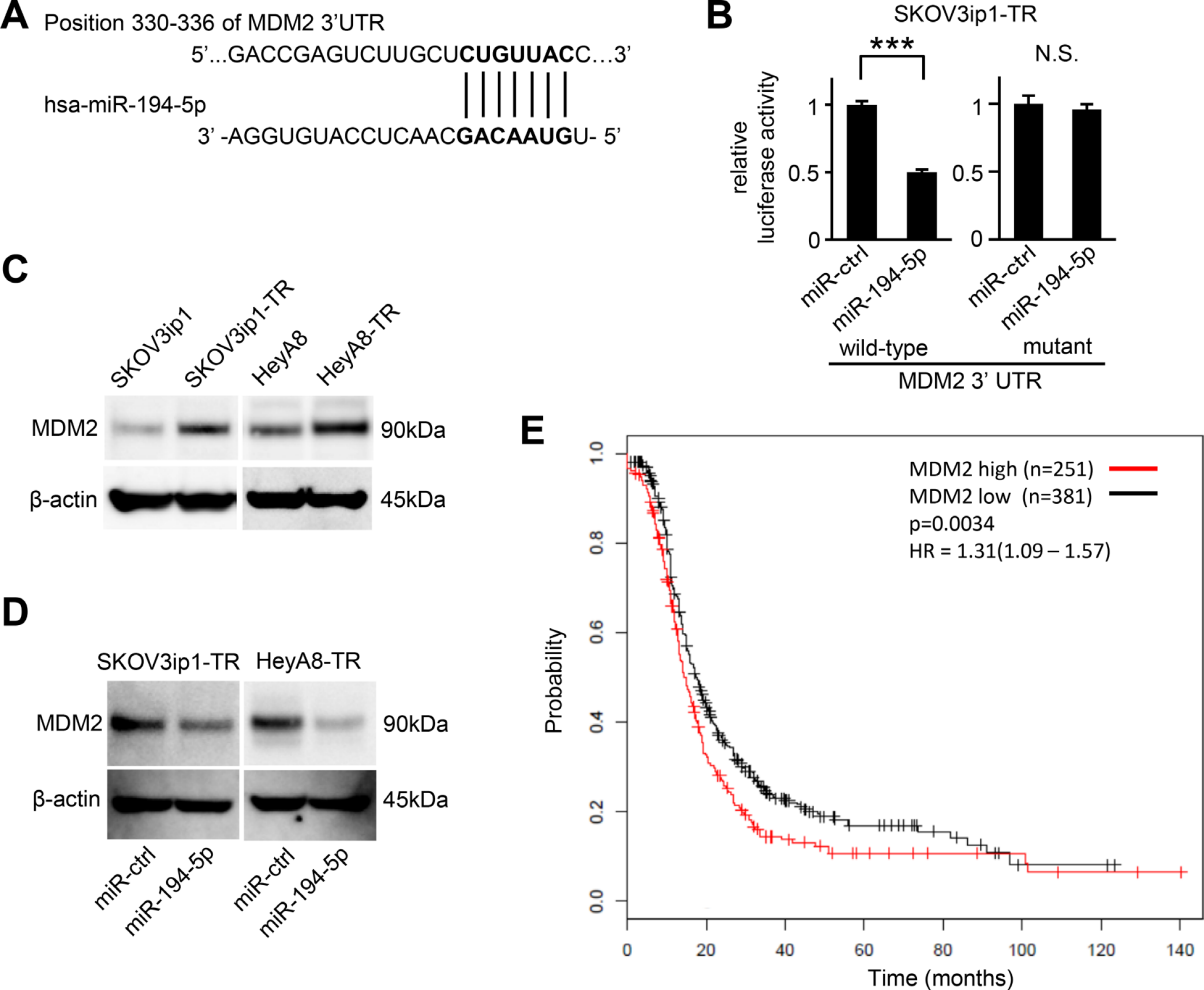
*MDM2* is a direct target gene of miR-194-5p and is upregulated in paclitaxel-resistant cells (**A**) Schematic illustration of predicted the *MDM2* 3′-UTR-binding site of miR-194-5p. (**B**) Luciferase reporter assay. SKOV3ip1-TR cells were co-transfected with miRNA precursor (miR-194-5p or miR-ctrl), luciferase reporter vector containing wildtype or mutant 3′-UTR of *MDM2*, and Renilla luciferase control vector. Twenty-four hours after incubation, luciferase activity normalized to Renilla activity was measured. Experiments were performed in triplicate. Data are represented as mean ± SE and are obtained from three independent experiments. ^***^P < 0.001; n.s., not significant. (**C** and **D**) Western blot. MDM2 expression between parental cell lines and paclitaxel-resistant sublines (C). After cells were transfected with miR-194-5p or miR-ctrl for 24 hours, cell lysates were collected and MDM2 expression was evaluated. β-actin was used as a loading control. Blots are representative of three experiments (D). (**E**) Kaplan-Meier survival analysis. Kaplan Meier Plotter database indicated that high *MDM2* expression in ovarian cancer patients treated with paclitaxel (*n* = 632) correlates with poor progression-free survival (*P* = 0.0034).

To determine how miR-194-5p downregulation is involved in paclitaxel resistance in ovarian cancer, putative targets of miR-194-5p were searched through *in silico* analyses. Among hundreds of putative target genes, we extracted genes related to chemoresistance and focused on *MDM2* as a candidate. TargetScan [9], and Diana Tools [10] revealed a putative miR-194-5p target site in the 3′-UTR of *MDM2* mRNA (position 330–336 of the *MDM2* 3′-UTR, Figure 3A). Thus, we constructed a pMIR-REPORT firefly luciferase miRNA expression reporter vector containing this putative miR-194-5p binding site for use in luciferase reporter assays. A significant decrease in relative luciferase activity was observed in SKOV3ip1-TR (0.50-fold; *P* < 0.001) cells transfected with miR-194-5p precursor as compared to those transfected with control miRNA (Figure 3B left). Conversely, site-specific mutation of the target sequence prevented the downregulation of luciferase activity induced by pre-miR-194-5p (Figure 3B right). These results indicated that miR-194-5p directly interacts with the *MDM2* 3ʹ-UTR. Western blot analysis revealed that paclitaxel-resistant cells showed a higher level of *MDM2* than their parental cell lines (Figure 3C). Moreover, the transduction of miR-194-5p in paclitaxel-resistant cells decreased *MDM2* expression (Figure 3D). To analyze the involvement of *MDM2* expression in paclitaxel resistance, a public database including the clinical information of 1436 ovarian cancer patients was analyzed [11]. Among 632 patients who were treated with paclitaxel, those with higher of *MDM2* expression showed significantly shorter progression-free survival (*P* = 0.0034, Figure 3E), suggesting that MDM2 might be related with paclitaxel resistance. Collectively, our data indicated that *MDM2* is a direct target of miR-194-5p and its expression is upregulated during the acquisition of paclitaxel resistance.

### MDM2 modulates sensitivity to paclitaxel

**Figure 4:**
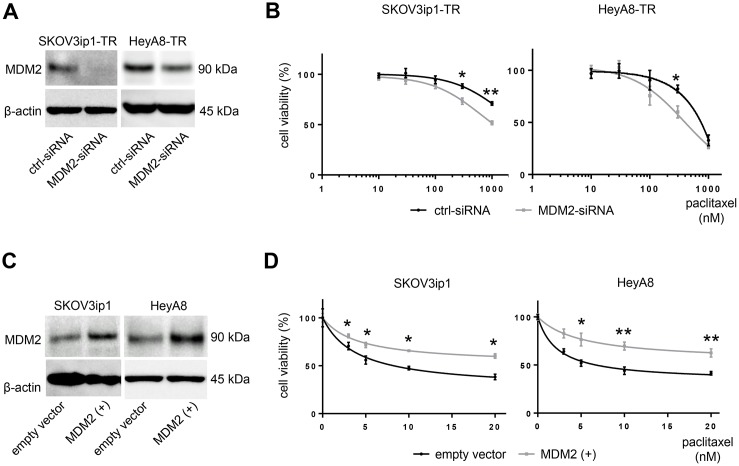
MDM2 modulates sensitivity to paclitaxel in ovarian cancer cell lines (**A**) Western blot. Cells were transfected with MDM2-siRNA or control-siRNA. Twenty-four hours after incubation, cell lysates were collected, and MDM2 expression was evaluated. (**B**) MTS assay. Cells were transfected with MDM2-siRNA or control-siRNA. Twenty-four hours after incubation, cells were treated with paclitaxel for 72 hours and cell viability was assessed. (**C**) Western blot. Cells were transfected with an *MDM2* expression plasmid (MDM2(+)) or empty vector. Forty-eight hours after transfection, cell lysates were collected. (**D**) MTS assay. Forty-eight hours after transfection with an *MDM2* expression plasmid (MDM2(+)) or empty vector, cells were treated with paclitaxel for 72 hours and viability was assessed. Blots are representative of three experiments. MTS assays were performed in triplicate. Data are represented as mean ± SE and are obtained from three independent experiments. ^*^P < 0.05, ^**^P < 0.01.

MDM2 is known as an oncogene that negatively regulates p21 as well as p53 [12, 13]. Previous reports have demonstrated that MDM2 mediates chemotherapy resistance [14]. To confirm the role of MDM2 in paclitaxel resistance, we tested cell viability upon paclitaxel treatment by either silencing or restoring MDM2. We transfected *MDM2*-targeting siRNA (MDM2-siRNA) or scramble-siRNA (ctrl-siRNA) into paclitaxel-resistant cells and transfected an *MDM2* expression vector or empty vector into parental cells. MDM2 knockdown or overexpression was confirmed at the protein level with western blotting (Figure 4A, 4C). MDM2 knockdown sensitized cells to paclitaxel. IC_50_ values for paclitaxel in SKOV3ip1-TR cells were > 1000 nM (ctrl-siRNA) and 816 nM (MDM2-siRNA), respectively, and they were 634 nM (ctrl-siRNA) and 440 nM (MDM2-siRNA), respectively in Hey-A8 cells (Figure 4B). Conversely, parental cells transfected with the *MDM2* expression vector showed greater resistance to paclitaxel than controls. IC_50_ values for paclitaxel in SKOV3ip1 cells were 7.35 nM (empty vector) and 24.6 nM (MDM2 vector), respectively. The IC_50_ values for paclitaxel in HeyA8-TR cells were 8.44 nM (empty vector) and 41.4 nM (MDM2 vector), respectively (Figure 4D). These results suggested that expression of not only miR-194-5p, but also its direct target *MDM2*, modulates paclitaxel sensitivity in ovarian cancer cells. This data also supports the hypothesis that miR-194-5p attenuates paclitaxel resistance through regulating MDM2 expression.

### miR-194-5p and its target MDM2 induce G0/G1 cell cycle arrest

**Figure 5:**
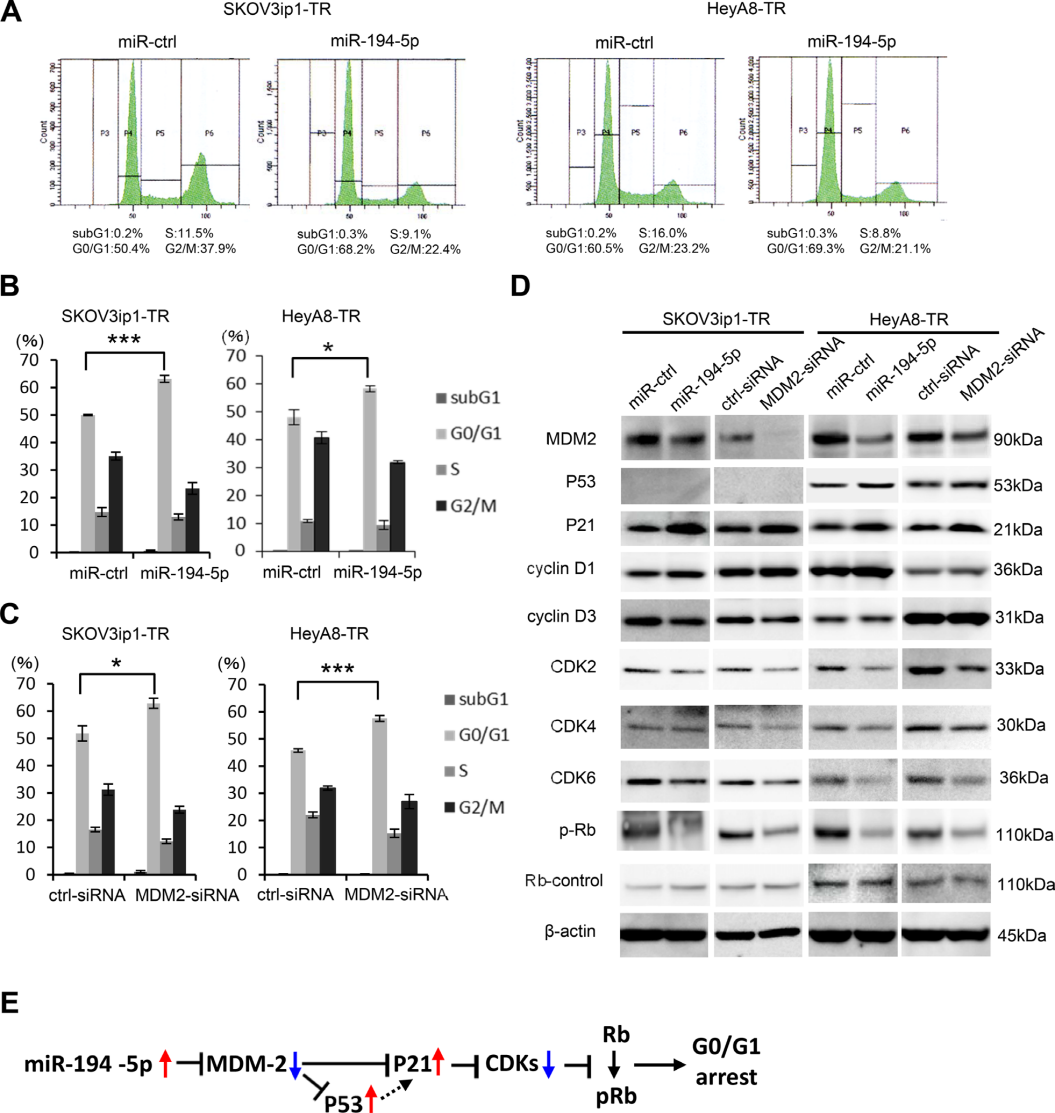
miR-194-5p and its target MDM2 induce G0/G1 cell cycle arrest (**A**–**C**) Cell cycle analyses. Paclitaxel-resistant cells transfected with miRNA precursors (miR-194-5p or ctrl-miR) or siRNAs (MDM2-siRNA or ctrl-siRNA) were stained with propidium iodide and analyzed by flow cytometry. Representative flow histograms. P3 = subG1 phase. P4 = G0/G1 phrase. P5 = S phase. P6 = G2/M phase (A). Percentage of cells in subG1, G0/G1, S, and G2/M phase. Paclitaxel-resistant cells transfected with miRNA precursors (B) or siRNAs (C). Data are represented as mean ± SE and are obtained from three independent experiments. (**D**) Western blot (left, SKOV3ip1-TR cells; right, HeyA8-TR cells). Expression of MDM2 and cell cycle-related proteins in paclitaxel-resistant cells transfected with miRNA precursors or siRNAs. Blots are representative of three experiments. (**E**) Schematic diagram of the pathway downstream of miR-194-5p. ^*^P < 0.05, ^***^P < 0.001.

Previous studies have shown that inhibiting MDM2 restores p53/p21 activity and subsequent cell cycle arrest in tumor cells [15]. Thus, we performed cell cycle analysis by flow cytometry to test the effect of miR-194-5p and MDM2 on the cell cycle. Transduction of miR-194-5p significantly increased the proportion of cells in the G0/G1 phase (in SKOV3ip1-TR: percent of G0/G1 phase, control miR vs miR-194-5p: 50.0 vs 63.1, *P* = 0.0005; in HeyA8-TR: percent of G0/G1 phase, control miR vs miR-194-5p: 48.2 vs 58.3, *P* = 0.03), and decreased the proportion of cells in S and G2/M phases in paclitaxel-resistant cells (in SKOV3ip1-TR: percent of G2/M phase, control miR vs miR-194-5p: 35.3 vs 23.1, *P* = 0.01; in HeyA8-TR: percent of G2/M phase, control miR vs miR-194-5p: 40.9 vs 32.0, *P* = 0.02) (Figure 5A, 5B), indicating that G0/G1 arrest was caused by miR-194-5p transduction. Similar results were observed when *MDM2* expression was inhibited in paclitaxel-resistant cells using siRNA (in SKOV3ip1-TR: percent of G0/G1 phase, scramble siRNA vs si-MDM2: 51.8 vs 62.9, *P* = 0.03; in HeyA8-TR: percent of G0/G1 phase, scramble siRNA vs si-MDM2: 45.8 vs 57.5, *P* = 0.0007) (in SKOV3ip1-TR: percent of G2/M phase, scramble siRNA vs si-MDM2: 31.2 vs 23.8, *P* = 0.04; in HeyA8-TR: percent of G2/M phase, scramble siRNA vs si-MDM2: 31.9 vs 26.9, *P* = 0.14) (Figure 5C). To further investigate the effect of miR-194-5p on cell cycle, the expression of cell cycle-related proteins was examined by western blot analysis using paclitaxel-resistant cells. miR-194-5p transduction and the silencing of MDM2 decreased MDM2 expression and thereby increased p53/p21 expression in p53 wild type HeyA8-TR cells [16] (Figure 5D, right). While it is known that SKOV3 is p53 null [17], p21 expression was increased after miR-194 or MDM2-siRNA transfection in SKOV3ip1-TR cells (Figure 5D, left). Although the expression of cyclins D1 and D3 did not remarkably change, the expression of multiple cyclin-dependent kinases such as CDK2, CDK4, CDK6, and phospho-Rb was also decreased upon miR-194-5p transduction and silencing of MDM2 (Figure 5D). Collectively, our data indicate that the restoration of miR-194-5p expression, which is downregulated during the acquisition of paclitaxel resistance, induces G0/G1 cell cycle arrest through signaling pathways downstream of MDM2, thereby attenuating paclitaxel resistance (Figure 5E).

## DISCUSSION

Overcoming chemoresistance is one of the major challenges in treating cancer. Chemoresistance is classified into two types: inherent resistance to the drug, and acquired resistance mediated by prolonged exposure to the drug. Given that the initial treatment of paclitaxel combined with platinum shows a high response rate (approximately 70%) in patients with advanced ovarian cancer [18, 19], paclitaxel remains the first-line chemotherapy for ovarian cancer treatment [20]. However, acquired resistance eventually occurs in the majority of patients, limiting the long-term efficacy of this drug. Indeed, the development of drug resistance is responsible for approximately 90% of the deaths among patients with advanced ovarian cancer [21]. Although several molecular mechanisms underlying paclitaxel resistance have been reported [22], these mechanisms are complex, involving multiple steps and multiple genes, and have not been yet fully elucidated. This is supported by the fact that no curative treatment option exists after paclitaxel resistance is acquired.

The accumulating evidence of miRNAs’ involvement in malignancy has provided new directions for research on mechanisms underlying response to chemotherapy. Several reports have focused on the functional role of miRNAs in paclitaxel resistance in ovarian cancer [23–25]. In this study, we established paclitaxel-resistant EOC lines by long-term exposure of paclitaxel to mimic acquired resistance and found that miR-194-5p was downregulated in paclitaxel-resistant cells. Restoring miR-194-5p in paclitaxel-resistant cells resensitized the cells to paclitaxel and inhibiting miR-194-5p in paclitaxel-sensitive parental cells decreased their sensitivity to paclitaxel. We suggested that *MDM2* is one of the direct target genes of miR-194-5p. Transducing miR-194-5p into paclitaxel-resistant cells reduced the level of MDM2 and downregulated cell cycle-associated molecules such as multiple cyclin-dependent kinases and phospho-Rb. These changes resulted in G0/G1 cell cycle arrest, which rendered the cells more sensitive to paclitaxel.

Our current understanding of the biological functions of miR-194-5p is still limited and somewhat inconsistent. Previous reports have shown that miR-194-5p has different expression patterns and plays opposite roles in different human cancers. For instance, Wang et al. demonstrated that miR-194-5p is commonly repressed in colorectal cancer and miR-194-5p overexpression regulates the MAPK4K/c-Jun/MDM2 signaling pathway and inhibits cell proliferation [6]. Wu et al. showed that miR-194-5p suppresses metastasis of non-small cell lung cancer by regulating BMP1 and p27kip1 [7]. Dong et al. showed that miR-194-5p inhibits the epithelial to mesenchymal transition by targeting BMI-1 and inhibits tumor invasion in endometrial cancer [26]. In contrast, Zhang et al. demonstrated that miR-194-5p is upregulated in pancreatic ductal adenocarcinoma compared with expression in adjacent normal pancreas tissue and that overexpression of miR-194-5p promotes cell proliferation, migration, and colony formation in pancreatic cancer cells [27]. Regarding ovarian cancer, one report by An et al. showed that miR-194-5p is downregulated in paclitaxel-resistant ovarian cancer tissue [8]. However, another report by Liang et al. described that miR-194-5p promotes cell growth, migration, and invasion by regulating PTPN12 [28]. These discrepancies suggest that the role of miR-194-5p in ovarian cancer is complicated and pleiotropic, and further research is needed to understand the entire miR-194-5p regulatory network.

Herein, we identified *MDM2* as a direct target of miR-194-5p and as a key regulator of paclitaxel resistance and MDM2 downregulation through miR-194-5p induced p21 expression, and subsequently that of cell cycle associated molecules regardless of the p53 status. As an E3 ubiquitin ligase, MDM2 plays an oncogenic role by blocking p53 transcriptional activity [29], and its overexpression frequently antagonizes p53 function in several types of cancers [30, 31]. Therefore, in p53 wild type HeyA8-TR cells [16], the transduction of miR-194-5p induced p53 accumulation through MDM2 downregulation. This in turn influenced the expression of the p53 target gene p21 and its downstream signaling pathway, and resulted in G0/G1 cell cycle arrest in paclitaxel-resistant ovarian cancer cells. MDM2 also directly facilitates p21 degradation independent of ubiquitination [13, 32]. In p53 null SKOV3ip1-TR cells, the transduction of miR-194-5p directly increased p21 expression through p53-independent mechanisms, which also resulted in G0/G1 cell cycle arrest in paclitaxel-resistant ovarian cancer cells.

Our data suggest that restoring miR-194-5p expression has the potential to overcome paclitaxel resistance. Although miRNA replacement therapy has been attempted in preclinical trials and many promising results have been reported, the outcomes of a few translational clinical trials for miRNA replacement therapy have been disappointing so far [33]. However, in 2017, van Zandwijk et al. reported a Phase I TargomiR trial with miR-16 mimics to treat patients with malignant pleural mesothelioma (NCT02369198) [34]. They found an acceptable safety profile and early signs of activity of TargomiRs and decided to enter a Phase II study. In this study, a bacterial-derived transfection method was used. Improving miRNA delivery systems by developing more specific carriers and manipulating epigenetic factors that enhance miRNA function will be the keys for the future success of miRNA replacement therapy [33].

In conclusion, in this study, we found that miR-194-5p loss is deeply involved in acquired resistance to paclitaxel and restoring miR-194-5p expression attenuates paclitaxel resistance by targeting *MDM2* in ovarian cancer cells. Although there is still a long way to go before the establishment of an effective and nontoxic miRNA replacement therapy targeting miR-194-5p, the results of this study suggest this strategy as a viable option for treating paclitaxel-resistant ovarian cancer that should be further explored for future clinical applications.

## MATERIALS AND METHODS

### Materials

Dulbecco's modified Eagle's medium (DMEM; #08458-45) was obtained from Nacalai Tesque (Kyoto, Japan). Fetal bovine serum (FBS; #172012) and paclitaxel (T7402) were purchased from Sigma Aldrich (St. Louis, MO). Antibodies against MDM2 (sc-965) and P53 (sc-126) were obtained from Santa Cruz Biotechnology (Dallas, TX). The cell cycle regulation antibody sampler kit (#9932), antibodies against β-actin (#4967), Rb-control (#9303), and phospho-Rb (#9307) were obtained from Cell Signaling (Danvers, MA). Lipofectamine 2000 Transfection Reagent (#11668027) and TRIzol Reagent (#15596-018) were from Life Technologies (Carlsbad, CA).

### Cell culture

The SKOV3ip1 cell line was generously provided by Dr. Ernst Lengyel (University of Chicago, IL). The HeyA8 cell line was generously provided by Dr. Anil Sood (MD Anderson Cancer Center, TX). Cells were cultured in DMEM supplemented with 10% FBS and 100 U/mL penicillin/streptomycin and incubated in 95% air/5% CO_2_ at 37°C. Cells were authenticated by short tandem repeat DNA profiling at Takara-Bio Inc. (Otsu, Japan) and were used for this study within 6 months of resuscitation.

### Establishment of paclitaxel-resistant ovarian cancer cell lines

Two paclitaxel-resistant sublines from SKOV3ip1 and HeyA8 were developed by stepwise increases in paclitaxel concentration from 1 nM to 150–300 nM over a period of 3 months. The resulting paclitaxel-resistant sublines, designated as SKOV3ip1-TR and HeyA8-TR, respectively, were cultured with 150–300 nM paclitaxel in the culture medium to maintain paclitaxel resistance.

### miRNA RT-qPCR array

Total RNA was collected from SKOV3ip1, SKOV3ip1-TR, HeyA8, and HeyA8-TR cells using TRIzol and were subjected to miRNA RT-PCR. For paclitaxel-resistant cells, RNA was collected after 48 hours of culture in the absence of paclitaxel. miRNA expression profiling was performed using the stem loop RT-qPCR-based TaqMan Human MicroRNA Array Set v2.0 (Applied Biosystems, Carlsbad, CA; #4398965) according to the manufacturer's protocol.

### RT-qPCR analysis of miR-194

RT-qPCR was performed using the StepOnePlus Real-Time PCR System (Applied Biosystems, Foster City, CA). Total RNA was extracted using TRIzol and transcribed into cDNA using the TaqMan MicroRNA Reverse Transcription Kit (Applied Biosystems; #4366596). Mature miR-194 was assayed using a TaqMan assay (hsa-miR-194-5p; #A25576). The TaqMan endogenous control (RNU6B; #001093) was used to normalize miRNA expression levels. Comparative real-time PCR was performed in triplicate, and relative levels of miR-194-5p expression were calculated using the 2^-ΔΔCt^ method [35].

### Transfection of miRNA

Ovarian cancer cells were transfected with precursor miRNA (pre-hsa-miR-194-5p, #PM10004) or inhibitor miRNA (anti-hsa-miR-194-5p, #AM10004) at a concentration of 50 nM. Pre-miR miRNA Precursor Negative Control #1 (#AM17110) was used as a control. All oligonucleotides were purchased from Thermo Fischer Scientific (Waltham, MA). Oligonucleotide transfection was performed using Lipofectamine 2000 according to the manufacturer's instructions. Twenty-four to 48 hours after transfection, cells were used for subsequent analysis.

### Cytotoxicity assay

Ovarian cancer cells (3 × 10^3^ cells/well) were seeded into 96-well plates. The cells were cultured for 24 hours prior to the addition of paclitaxel. Paclitaxel was diluted to a range of concentrations (1–1000 nM) in DMEM substituted with 2% FBS and was added to the cells; cells were incubated for 72–96 hours. Thereafter, 20 μL of MTS (3-(4,5-dimethylthiazol-2-yl)-5-(3-carboxymethoxyphenyl)-2-(4-sulfophenyl)-2H-tetrazolium, inner salt) (Promega, Fitchburg, WI) solution was added to the cells, which were then incubated at 37°C for 1 h. The optical density was measured at 490 nm. Relative cell viability was normalized to viability in paclitaxel-free conditions. IC_50_ values were calculated using the GraphPad Prism 7 software (GraphPad Software, San Diego, CA).

### Western blotting

Cells were lysed using 1x Cell Lysis Buffer (20 mM Tris-HCl (pH 7.5), 150 mM NaCl, 1 mM Na_2_EDTA, 1 mM EGTA, 1% Triton X-100, 2.5 mM sodium pyrophosphate, 1 mM beta-glycerophosphate, 1 mM Na_3_VO_4_, 1 μg/ml leupeptin; Cell Signaling). Cell lysates were separated by SDS-PAGE using SuperSep Ace 5–20% (Wako, Osaka, Japan) and transferred to polyvinylidene difluoride membranes. The membranes were then incubated with the appropriate primary antibody (anti-β-actin (1:1000), anti-MDM2 (1:1000), anti-P53 (1:1000), anti-P21 (1:1000), anti-Cyclin D1 (1:1000), anti-Cyclin D3 (1:1000), anti-CDK2 (1:250), anti-CDK4 (1:250), anti-CDK6 (1:250), anti-Rb-control (1:250), anti-phospho-Rb (1:250)) diluted in TBS with 5% BSA, followed by incubation with a corresponding secondary horseradish peroxidase conjugated IgG at a dilution of 1:10,000. The proteins were visualized using an ECL system (PerkinElmer Life Science, Boston, MA), and luminescent images were analyzed by a Luminoimage analyzer (ImageQuant LAS-4000; GE Healthcare Bio-Sciences Corp., Piscataway, NJ).

### Cell cycle analysis

Cells (2 × 10^5^) were plated onto 6-well dishes and transfected with oligonucleotides. Twenty-four to 48 h after transfection, cells were collected and fixed overnight in 75% ethanol. Then, cells were stained with 50 μg/ml propidium iodide (P4864, Sigma) in the presence of 100 μg/ml RNase A (Roche) for 1 h at 4°C. Cell cycle distribution was determined by analyzing 1 × 10^4^ cells using a BD FACSCanto II flow cytometer (BD, Franklin Lakes, NJ). Data analysis was performed using BD FACSDiva software (BD).

### Luciferase reporter assay

Synthetic oligonucleotides with 4 copies of the MDM2 3′-UTR (GACCGAGTCTTGCTCTGTTACCC; bp 316–338 of ENST00000462284), which were predicted to bind with hsa-miR-194-5p, or 4 copies of a mutated version of the sequence (GAGCGTGTCTTGCTCTCTTTCCC) with MluΙ and XhoΙ restriction sites at each end, were cloned into the pMIR-REPORT firefly luciferase miRNA expression reporter vector (Applied Biosystems; #AM5795). After 5 × 10^4^ ovarian cancer cells were seeded onto 24-well plates, 0.5 μg of the pMIR-REPORT vector, 0.05 μg of the pRL-TK Renilla luciferase control vector (Promega, Madison, WI, USA; #E2241), and 40 pM pre-miR-194-5p or the negative control miRNA were co-transfected using Lipofectamine 2000. Twenty-four hours after transfection, luciferase activity was measured using the Dual-Luciferase Reporter Assay System (Promega; #E1910) according to the manufacturer's instructions. Firefly luciferase activities were normalized to Renilla luciferase activities.

### Analysis of the prognostic value of *MDM2* using a public database

The prognostic value of *MDM2* gene expression was determined in 632 patients with serous ovarian cancer who were treated with paclitaxel, using the Kaplan-Meier Plotter, which is a database that integrates gene expression data and clinical data [12]. The patient samples were split into two groups (high or low *MDM2* expression) automatically and compared using a Kaplan-Meier survival plot. Hazard ratios with 95% confidence intervals and log rank *p* values were calculated.

### Statistical analysis

JMP Pro 11 (SAS Institute Japan Ltd., Tokyo, Japan) was used for statistical analysis.

Except for RT-qPCR data, all data were expressed as mean ± SEM. For RT-qPCR data, the error bars represent standard deviation calculated by the StepOnePlus Real-Time PCR System. Differences were analyzed using the Mann-Whitney *U* test. To analyze the correlation between two variables, the Pearson's product-moment coefficient of correlation and associated *P* value were calculated. Differences were considered statistically significant at *P* < 0.05.

## Abbreviations

EOC: epithelial ovarian cancer
miRNA: microRNA
